# Exploiting Spatial Ionic Dynamics in Solid‐State Organic Electrochemical Transistors for Multi‐Tactile Sensing and Processing

**DOI:** 10.1002/advs.202405902

**Published:** 2024-09-27

**Authors:** Kunqi Hou, Shuai Chen, Rohit Abraham John, Qiang He, Zhongliang Zhou, Nripan Mathews, Wen Siang Lew, Wei Lin Leong

**Affiliations:** ^1^ School of Physical and Mathematical Sciences Nanyang Technological University 21 Nanyang Link Singapore 637371 Singapore; ^2^ School of Electrical and Electronic Engineering Nanyang Technological University 50 Nanyang Avenue Singapore 639798 Singapore; ^3^ Laboratory of Inorganic Chemistry Department of Chemistry and Applied Biosciences ETH Zürich Zürich CH‐8093 Switzerland; ^4^ School of Materials Science and Engineering Nanyang Technological University 50 Nanyang Avenue Singapore 639798 Singapore

**Keywords:** in‐electrolyte computing, ion modulation, organic electrochemical transistor, self‐multiplexer platform, solid‐state, tactile sensors

## Abstract

The human nervous system inspires the next generation of sensory and communication systems for robotics, human‐machine interfaces (HMIs), biomedical applications, and artificial intelligence. Neuromorphic approaches address processing challenges; however, the vast number of sensors and their large‐scale distribution complicate analog data manipulation. Conventional digital multiplexers are limited by complex circuit architecture and high supply voltage. Large sensory arrays further complicate wiring. An ʻin‐electrolyte computingʼ platform is presented by integrating organic electrochemical transistors (OECTs) with a solid‐state polymer electrolyte. These devices use synapse‐like signal transport and spatially dependent bulk ionic doping, achieving over 400 times modulation in channel conductance, allowing discrimination of locally random‐access events without peripheral circuitry or address assignment. It demonstrates information processing from 12 tactile sensors with a single OECT output, showing clear advantages in circuit simplicity over existing all‐electronic, all‐digital implementations. This self‐multiplexer platform offers exciting prospects for circuit‐free integration with sensory arrays for high‐quality, large‐volume analog signal processing.

## Graphical abstract

An ‘in‐electrolyte computing' platform is demonstrated using spatially modulated ionic doping in all‐solid‐state organic electrochemical transistors. Over 400‐fold modulation is achieved using this approach, enabling precise differentiation of 12 tactile signals with a single polymeric channel. This approach significantly simplifies circuit design and reduces the use of electrical components.

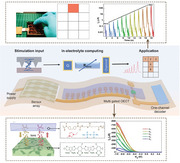

## 1 Introduction

The human nervous system provides a foundational framework for advancing sensory processing and communication systems in robotics, human‐machine interfaces (HMIs), biomedical technologies, and artificial intelligence. While neuromorphic approaches address processing challenges, signal multiplexing of analog inputs is always overlooked.^[^ ^1^, ^2^, ^3^, ^4^ ^]^ Conventional electronic multiplexers have limited logic states, complex circuit architecture, and high supply voltage, leading to issues in space, cost, and power consumption.^[^ ^5^, ^6^ ^]^ Additionally, they suffer from crosstalk effects between neighboring wires, reducing the signal‐to‐noise ratio (SNR).^[^ ^7^, ^8^ ^]^ Due to the analog nature of sensory data, an analog‐digital converter is needed for digital transistors. However, as data processing workloads increase, an integral device capable of directly handling and distinguishing analog signals with added circuit simplicity is highly desired.^[^ ^9^ ^]^

Organic electrochemical transistors (OECTs) are promising candidates for analog signal multiplexing. These devices operate by harnessing the electrochemical doping effect of conjugated polymers, where dopant ions from the electrolyte penetrate the polymer network, altering its doping state and switching the transistor between ON and OFF states.^[^ ^10^, ^11^, ^12^ ^]^ Their unique structure offers an interactive interface between biology and electronics through bulk ionic‐electronic interactions, making them attractive for applications ranging from neuromorphic devices,^[^ ^13^, ^14^ ^]^ logic circuits,^[^ ^15^, ^16^, ^17^, ^18^, ^19^ ^]^ and bioelectronic sensors.^[^ ^20^, ^21^, ^22^ ^]^ In particular, by carefully tuning the electrolyte impedance, non‐linear coupling of resultant potentials within the electrolyte enables multi‐state channel conductance and different ion and electron dynamics inside the conjugated semiconductors,^[^ ^23^, ^24^ ^]^ enabling ionic multiplexing, referred to as “in‐electrolyte computing”.^[^ ^25^, ^26^, ^27^ ^]^

The use of ʻin‐electrolyteʼ computing’ is particularly useful for enhancing the functionality and applicability of OECTs, facilitating advancements in areas such as ionic multiplexing,^[^ ^27^ ^]^ robotic control,^[^ ^26^ ^]^ and neuromorphic computing.^[^ ^28^ ^]^ However, previous efforts that utilize liquid electrolytes have faced limitations due to their restricted working temperature range and unsuitability for further electronic packaging due to the risk of liquid evaporation/leakage.^[^ ^29^ ^]^ On the other hand, liquid electrolytes have demonstrated a weak ion modulation effect in OECTs with less than 10 modulation ratio (I_max_/I_min_), leading to reduced addressing accuracy due to the inevitable overlap of neighboring conducting states and a limited number of recognizable input signals (< 5 signals). This weak ion modulation is possibly attributed to high degree of ion mobility within the liquid electrolyte, resulting in a diminished spatially dependent ionic impedance modulation.

Here, we present an ionic analog multiplexer platform utilizing a single all‐solid‐state OECT (SSOECT). Compared with previously reported liquid‐based ionic modulators, our solid electrolyte exhibits superior long‐term stability (>90% data retention for 2 weeks under air ambient) and thermal stability.^[^ ^30^ ^]^ More importantly, we achieved a modulation ratio exceeding 400‐fold, ≈100 times higher compared to reported ion‐modulated multiplexers^[^ ^26^, ^27^ ^]^) through fine‐tuning of the ionic liquid concentration for a more controlled ionic movement within the solid electrolyte, and investigating the temporal response of SSOECTs. The high modulation ratio in the dynamic transient regime enables our device to differentiate between 12 distinct physical tactile input signals under a low voltage bias (less than 1.5 V). Additionally, the amplitude of the output can be analogously adjusted based on the applied incident pressure, and external tactile signals applied to each sensor can be translated to their corresponding OECT channel current ʻsignatureʼ. This time‐varying signal demonstrates more complex functions with reduced circuit complexity.

## 2 Results and Discussion

**Figure 1:**
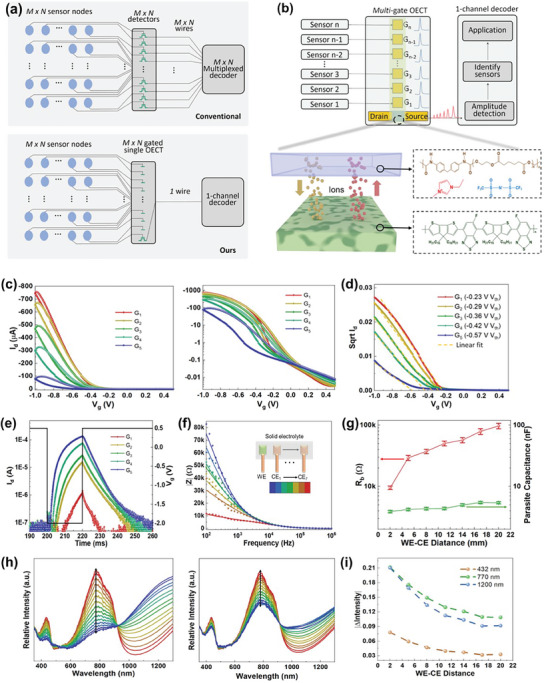
Working principle of the proposed ʻin‐electrolyte computingʼ platform. a) Comparison of conventional multiplexing schemes and our synapse‐like analog multiplexer. b) Schematic of a 5‐gate self‐multiplexing SSOECT, including the materials and chemical structures of the electrolyte polymer matrix, ionic liquid, and semiconductor used in this study. The distances between gates 1 and 5 and the channel are 1, 3, 6, 10, and 15 mm, respectively. The solid electrolyte covers the entire channel and gate area, isolated by a 1.4 µm‐thick parylene layer. Solid electrolytes containing 37.5 wt.% ionic liquid and transistor channels with W/L = 100/10 µm were used for electrical characterizations unless otherwise noted. The thickness of the spin‐coated semiconductor film is 81 nm, and all gates have a size of 600 µm × 600 µm. c) Transfer performance of the transistor for each gate with varying spatial dynamics, shown in linear and semi‐log scales. d) Shift of Vth with varying spatial dynamics of gates due to changes in V_eff_. Threshold voltages were extracted from the intercept of the *x*‐axis. e) Transient behavior of multiterminal SSOECT measured with a rectangular pulse of 20 ms/−2 V pulse width/amplitude. f) Impedance spectrum amplitude (|Z| vs frequency) for varying CE‐WE distances. Measured data are shown with scatters, while the fitted curves using the equivalent circuit are shown with solid lines. g) Calculated bulk resistance and capacitance values of solid electrolytes at varying CE‐WE distances. h) Dynamic shifting of absorption spectra within 5 s under a −3 V gate bias on CE_1_ and CE_7_. i) Dynamic shift of absorption intensity at 432, 772, and 1200 nm at varying CE‐WE distances.

**Figure** **1**a compares conventional multiplexing schemes with our synapse‐like analog multiplexer. Traditional methods necessitate M×N transistors to detect input stimuli from M×N sensors. In contrast, our ionic‐multiplexing scheme employs a single M×N‐gated transistor with a polymeric channel, significantly reducing the number of electrical components. More importantly, the spatial‐dependent ionic movement within solid electrolytes achieves a one‐channel output, thereby minimizing circuit complexity and wire interconnections. Figure 1b and Figure S1 (Supporting Information) present the schematic and optical images of the fabricated ionic‐electronic multiplexer. This system incorporates a conjugated semiconducting channel composed of poly [(5‐fluoro‐2,1,3‐benzothiadiazole‐4,7‐diyl) (4,4‐dihexadecyl‐4H‐cyclopenta [2,1‐b:3,4‐b'] dithiophene‐2,6‐diyl) (6‐fluoro‐2,1,3‐benzothiadiazole‐4,7‐diyl) (4,4‐dihexadecyl‐4H‐cyclopenta [2,1‐b:3,4‐b'] dithiophene‐2,6‐diyl)] (PCDTFBT). This material functions as an enhancement‐mode p‐type semiconductor, where anions dope the polymer and induce additional holes under a negative gate bias, thereby enhancing conductivity.^[^ ^31^, ^32^ ^]^ The ionic liquid 1‐Ethyl‐3‐methylimidazolium bis(trifluoromethyl sulfonyl) amide (EMIM:TFSI) and a thermoplastic polyurethane (TPU) polymer matrix was selected to form the polymer electrolyte used as the communication medium of the multiplexer. TPU provides favorable mechanical and ionic properties for the electrolyte, while the hydrophobic TFSI^−^ anion can readily dope PCDTFBT under a negative bias, resulting in a lower threshold voltage.^[^ ^30^, ^33^ ^]^

In this work, the mechanism to realize ionic modulation within SSOECTs involves intricate control of the channel conductance via 5 side gates, which are in direct contact with the solid electrolyte, and are spaced at intervals ranging from 1 to 15 mm along the channel. This arrangement allows for differential ionic resistance (R_b_) across the channel depending on the gate‐to‐channel distance. A longer gate‐channel distance typically induces higher R_b_ within the solid electrolyte,^[^ ^27^ ^]^ leading to a greater voltage drop across it.^[^ ^34^ ^]^ Such spatially varying R_b_ directly impacts the ion movement and ionic‐electronic coupling within the channel. As ions traverse through these varying resistance zones, they experience different rates of penetration into and out of the conjugated polymer, effectively altering the doping state of the semiconductor. This in turn influences the effective gate voltage (V_eff_), allowing for localized modulation of channel conductivity and analogous current output (Figure 1b).^[^ ^23^, ^28^ ^]^ As shown in Figure 1c, the drain current (I_d_) correlates well with the spatial dynamics of the gates and changes in V_eff_. For instance, as the gate‐channel distance varies from 1 to 15 mm, a significant shift in the threshold voltage (V_th_) from −0.45 to −0.78 V is observed (Figure 1d). Moreover, the change in R_b_ also affects the characteristic charging time (τ = R_b_ × C).^[^ ^35^ ^]^ As the gate‐channel distance increases, the higher ionic resistance induces inefficient charging of the gate capacitor under the applied gate voltage (V_g_), resulting in a greater delay in ion penetration into the channel. The gate current (I_g_) shows a similar trend, being negatively proportional to the increasing gate‐channel distance (Figure S2, Supporting Information). Under temporal measurement protocols, the delayed increment in I_d_ was found to be more pronounced, resulting in a modulation ratio approximately ten times higher than that observed in steady‐state measurements, as shown in Figure 1e. Additionally, the use of solid electrolytes enables the long‐term operation of the OECT in ambient air conditions, with over 90% of the on‐current/modulation ratio retained and negligible change in the off‐current observed after two weeks (Figure S3, Supporting Information).

Subsequently, the change in impedance dynamics induced by the spatial distribution of gate electrodes was further investigated using electrochemical impedance spectroscopy (EIS) measurements. Different distances between the working electrode (WE) and counter electrode (CE) were examined, ranging from 2 to 20 mm (Figure S4, Supporting Information). The ion movement within the solid electrolyte can be modeled as a bulk ionic resistor (R_b_) in parallel with an ionic capacitor (Q_CPE2_). The R_b_ of the electrolyte can be tuned and shows a positive correlation with the distance between the WE and CEs, as illustrated in Figure S5 (Supporting Information).^[^ ^36^, ^37^ ^]^ In the R_b_ ‐Q_CPE2_ parallel circuit, ionic capacitance dominates at high frequency (Z = 1/Q(jw)^α^) and shows negligible spatial dependence (≈45 Ω at 1 MHz). In stark contrast, a substantial increase in impedance at low frequencies was observed, corresponding to the increment in R_b_ (Figure 1f; Figure S6, Supporting Information). The extracted ionic resistors and capacitors from the equivalent circuit are shown in Figure 1g. The R_b_ increased by one order of magnitude with an 18 mm change in the CE‐WE distance, while the ionic capacitance remained constant.

The in situ spectrochemical response of PCDTFBT films interfaced with TPU electrolyte also corroborates our findings on the gate‐channel distance‐modulated ionic doping effect (Figure S7, Supporting Information). The pristine polymer films exhibit an absorption peak at 432 nm, corresponding to *π*–*π* ^*^ transitions, followed by peaks extending into the IR range (772 and 1200 nm) due to intramolecular charge transfer (ICT) excitation (Figure S8, Supporting Information), which aligns well with previous reports.^[^ ^40^, ^41^ ^]^ As shown in Figure 1h and Figure S9 (Supporting Information), the application of a negative bias results in the injection of TFSI^⁻^ anions into the polymer layer and doping of the polymer. A more significant reduction in peaks at 432 and 772 nm was observed for smaller CE‐WE distances due to a larger V_eff_. The contrast in ionic doping dynamics between CE‐WE distances of 2 and 20 mm is particularly notable (Video S1, Supporting Information). The change in absorption intensity for different WE‐CE distances at 432, 772, and 1200 nm is further summarized in Figure 1i and Figure S10 (Supporting Information). The slope of intensity shift versus time correlates with τ and exhibits a positive relationship with the distance of ionic‐electronic coupling.^[^ ^38^ ^]^

**Figure 2:**
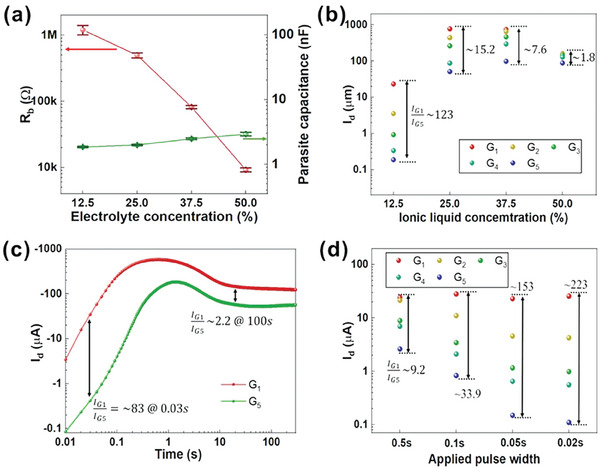
Investigation of parameters (ionic liquid concentration, channel dimension, channel material, and measurement protocol) influencing the speed and modulation ratio of the multiplexer. a) Calculated bulk resistance and capacitance values of solid electrolytes containing 12.5, 25, 37.5, and 50 wt.% ionic liquid at a 2 mm gate‐semiconductor distance. b) Extracted I_d_ for five gates from transfer curves under −1 V applied V_g_ with varying electrolyte concentrations. c) Output behavior of the local and remote gates under −1.4 V applied V_g_ for 5 min. d) Extracted I_d_ for five gates from transient curves under varying AC measurement protocols (PW/Amplitude = 0.5s/−0.6 V, 0.1s/−1 V, 0.05s/−1.2 V, and 0.02s/−2 V).

The communication between the gates and the channel is closely related to the ionic conductivity of the solid electrolytes. A decrease in the ionic liquid content within solid electrolytes significantly enhances their ionic resistance, providing a means to further modulate ion dynamics (**Figure** **2**a; Figure S11, Supporting Information). Consequently, a notable shift in V_eff_ is observed in OECTs utilizing solid electrolytes with low ionic liquid content under the same gate‐channel distance variation, leading to a more pronounced discrepancy in the OECT output current and yielding a larger modulation ratio (I_G1_/I_G5_ ≈123 with 12.5 wt.% ionic liquid, where I_G1_​ and I_G5_ denote the output current of the transistor when voltage is applied to G_1_ and G_5_, respectively), as depicted in Figure 2b. In contrast, OECTs employing solid electrolytes with 50 wt.% ionic liquid exhibits a subdued ion modulation effect due to their higher ionic conductance, resulting in a modest 1.8‐fold modulation ratio. Additionally, larger channels (W/L = 200/50 µm) were characterized for comparison. The larger channel area reduces channel impedance, accentuating the discernibility of electrolyte impedance changes and facilitating larger modulation ratios compared to OECTs with smaller channels (Figure S12, Supporting Information). However, OECTs utilizing solid electrolytes with low ionic content or larger channel areas exhibit significant hysteresis in transfer characteristics and slower response times due to sluggish ion mobility and substantial charging capacitance (Figure S13, Supporting Information).

We next shift our focus to the dynamic regime, when time‐dependent voltages are applied and varying on a time scale comparable to the τ of OECT (typically ranging in tens of millisecond),^[^ ^39^ ^]^ the ionic (dis)charging time must be taken into account.^[^ ^40^ ^]^ More specially, when I_d_ reaches saturation, the modulation ratio is primarily influenced by changes in V_eff_. However, by shortening the pulse width (PW) before the current saturation, an additional increase in the modulation ratio will be observed, which is attributed to the difference in charging speeds of varying gates. For instance, G_1_, with its low ionic resistance, facilitates faster ionic charging (τ = R_b_ × C), resulting in more efficient doping of the conjugated polymer and an earlier increase in I_d_ compared to G_5_, as demonstrated in Figure 2c. Due to this phenomenon, a more pronounced discrepancy in the OECT output current is observed before the saturation of G_5_ (I_G1_/I_G5_ ≈83 at 0.03 s), whereas the ratio is only ≈2.2 at 100 s, well beyond the saturation point. In this regard, the multiplexer was tested under varying measurement protocols, with pulse widths ranging from 20 to 500 ms. As expected, the highest modulation ratio in I_d_ of ≈223 was observed at a 20 ms pulse width, reflecting the frequency‐dependent property (see Figure 2d; Figure S14, Supporting Information). This observation allows us to further improve the modulation ratio without modifying the device structure and materials. Additionally, we explored the applicability of this multiplexing scheme to poly(3,4‐ethylenedioxythiophene)‐poly (styrene sulfonate) (PEDOT: PSS, depletion mode p‐type) and poly(3‐hexylthiophene‐2,5‐diyl) (P3HT, enhancement mode p‐type) channels, both of which exhibited a distance‐dependent ionic doping effect (Figure S15, Supporting Information).

**Figure 3:**
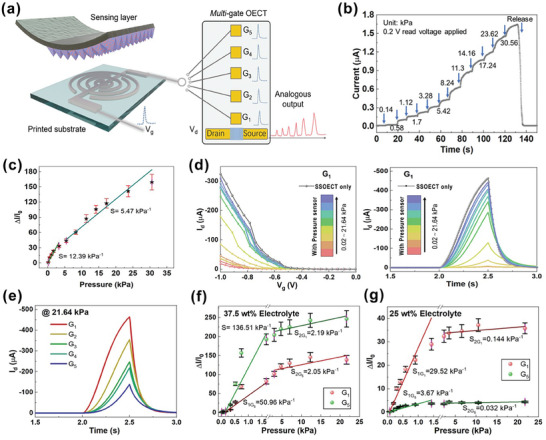
Integration of our analog multiplexer with the pressure sensor. Solid electrolytes with 37.5 wt.% ionic liquid were used. a) Schematic of the micro‐patterned pressure sensor and its integration with the analog multiplexer. b) Output behavior of the pressure sensor from 0.14 to 30.56 kPa under a 0.5 V read voltage. c) Sensitivity of the pressure sensor, extracted by deriving the slope in linear sensing regions. d) Transfer/transient behavior of the integrated system from 0.02 to 21.64 kPa. The pressure sensor is connected to G_1_ with −1.5 V/−0.5 V V_g_/V_d_ applied. e) Transient response of gates with varying spatial dynamics under 21.64 kPa. f,g) Comparison of sensitivities in the low and high‐pressure regions when the pressure sensor is connected to G_1_ and G_5_. OECTs with 37.5 wt.% solid electrolytes for and 25%.

To investigate the capability of our multiplexing platform for processing analog signals, we connected a pressure sensor to each gate terminal of the SSOECTs, as illustrated in **Figure** **3**a. The pressure sensor consists of micro‐pyramid‐patterned PEDOT: PSS on a styrene‐ethylene‐butylene‐styrene (SEBS) substrate. When no pressure is applied, the sensor exhibits a small current flow (≈10 nA) due to the minimal contact area between the PEDOT: PSS pyramids and the two silver electrodes. In contrast, when pressure is applied, the contact area and the compressed thickness of the pyramid structure increase, leading to a decrease in contact resistance and a subsequent increase in current, as demonstrated in Figure 3b. By deriving the slope in the linear sensing regions, we obtained pressure sensitivities of up to 9.142 kPa⁻¹ in the low‐pressure region (< 4 kPa) and 4.597 kPa⁻¹ in the high‐pressure region (4–30 kPa), as shown in Figure 3c.

Subsequently, the pressure sensor was sequentially connected from G_1_ to G_5_ of the multi‐gated SSOECT. Higher pressure induces greater gate‐modulation behavior, resulting from the higher V_eff_ applied to the SSOECT. This allows external pressure to trigger and control ionic penetration in the conjugated semiconductor, thereby modulating its conductivity. This demonstrates the successful detection of analog stimuli by the SSOECT‐based multiplexer, as shown in Figure 3d. Under the same applied bias on the pressure sensor, G_1_ through G_5_ exhibit a well‐defined spatial‐temporal response, indicating effective discrimination of random stimulation events at different locations, as shown in Figure 3e and Figure S16 (Supporting Information). By connecting the pressure sensor with OECTs, a significant improvement in sensitivity and sensing range was observed in the low‐pressure region (≈15 times higher than with a single pressure sensor), attributed to the high transconductance of OECTs, which amplifies small shifts in input dynamics through bulk ionic‐electronic coupling (Figure 3f). Additionally, we noted that the sensitivity of our system displayed an inverse correlation with the distance between the gate and channel (Figure S17, Supporting Information). This phenomenon is due to the larger ionic resistance generated by G_5_ on the OECT, which reduces the relative ratio of input impedance under varying incident pressures. Similar results were observed with OECTs using lower‐concentration solid electrolytes, which have larger ionic impedance (Figure 3g). These findings are consistent with our previous electrochemical impedance spectroscopy analysis in Figures 1g and 2a.

These results underscore the advantages of our multiplexer in directly amplifying and identifying analog signals from sensors without the need for an analog‐to‐digital converter (ADC), which is required in traditional implementations. Furthermore, the sensitivity of our system can be adjusted by engineering the electrolyte impedance. By carefully selecting solid electrolytes, it is possible to simultaneously determine both the magnitude and location of input stimuli using a single polymeric channel.

**Figure 4:**
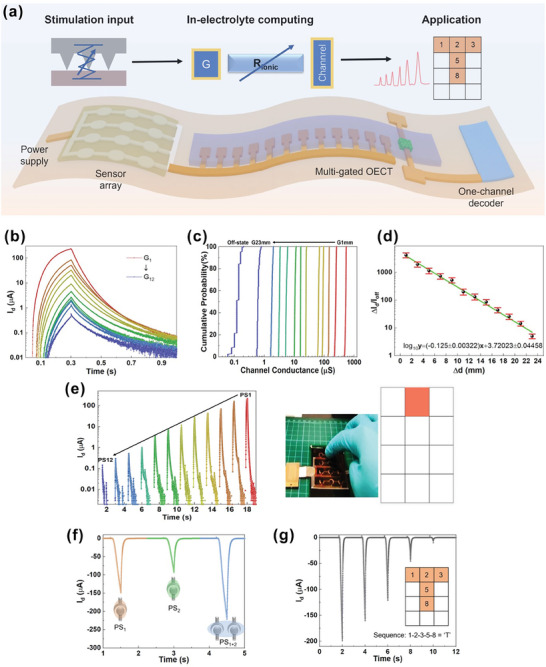
Application of the SSOECT‐based multiplexer for array tactile signal detection. The distances between gates 1 and 12 and the channel are 1, 3, 5, 7, 9, 11, 13, 15, 17, 19, 21, and 23 mm, respectively. Solid electrolytes with a 37.5 wt.% ionic liquid ratio was used. a) Schematic representation of the integrated system. b) Transient behavior of the 12‐gate SSOECT measured with a 300 ms pulse and −1.2 V amplitude. c) Cumulative probability of channel conductance over 100 cycles for all gates. d) Modulation ratio in drain current versus changes in distance. e) Current output of the multiplexer when stimulated with an identical tactile sensor. f) Current output of the multiplexer when pressing single or multiple tactile sensors. g) Pattern recognition achieved by the integrated system through the output current sequence from multiple sampling cycles.

To further demonstrate the advantages of this signal multiplexing technique for detecting multiple biological stimuli, 12 tactile sensors were integrated into a single SSOECT with 12 gate terminals via parallel connection (each individual sensor in series with its corresponding gate). This setup allows each sensor to trigger the SSOECT with varying amplitudes based on the spatial dynamics of the gates, as shown in **Figure** **4**a. Optical images of the custom‐made measurement setup are provided in Figures S18 and S19 (Supporting Information). To accurately and efficiently obtain spatial information of incident simulations applied on pressure sensors, a high modulation ratio and rapid recovery of the channel are essential. To achieve a high modulation ratio, a lower ionic liquid content is preferred, where the ionic penetration and accumulation within the polymer matrix can be more effectively modulated, facilitating precise sensing. However, the increased ionic resistance significantly extends the withdrawal time of dopant ions from the conjugated polymer, leading to incomplete recovery of the channel conductance and resulting in the reduction in sensing efficiency. We thus selected a 37.5% electrolyte concentration as it offers an optimal balance between high modulation ratio and adequate recovery speed (Figure S20, Supporting Information).

As depicted in Figure 4b, isolated voltage pulses were applied to each gate individually to activate the SSOECT‐based multiplexer. An excellent modulation ratio of ≈402‐fold (I_G1_/I_G12_) was observed, as illustrated in Figure 4b. To verify reliability, this stimulation procedure was repeated 100 times from G_12_ to G_1_, with no overlap between the resistance states of different gates, achieving 100% addressing accuracy, as shown in Figure 4c and Figure S21 (Supporting Information). Additionally, due to the exponential relationship in capacitor charging, the tuning ratio of channel conductance (Δ*I*/I_off_) decreases exponentially with increasing gate‐channel distance (Figure 4d).

During the experiment, when an input pressure stimulus is applied to an individual sensor (PS_n_), its corresponding gate (G_n_) is activated. This activation results in noticeable differences in the response amplitude of I_d_, which serves as unique “signatures” and facilitates information transmission, as shown in Figure 4e, Videos S2 and S3 (Supporting Information). In conventional implementations, a resistor ladder comprising 12 resistors and 12 transmission gates, incorporating 24 transistors, is necessary to distinguish input signals from 12 distinct sensors. In contrast, our approach utilizes the in‐electrolyte computing concept, reducing the number of electrical components to just one transistor.

We also investigated the simultaneous activation of two sensors, observing a larger increment in I_d_ (Figure 4f). This increase is likely due to the larger gate area, which reduces the gate‐electrolyte impedance, thereby allowing more anions to migrate into the channel. Additionally, this integrated system enables the monitoring of input finger motion patterns, where different patterns or letters generate distinct output current sequences from multiple sampling cycles (Figure 4g; Video S4, Supporting Information). To achieve the recognition of more complicated input pressure patterns within one voltage pulse, multiple output channels with distinct spatial dynamics can be used to obtain more detailed analog output information from various pressure patterns. These demonstrations highlight the potential of our technique for constructing functional devices without complex circuitry and interconnections.

## 3 Conclusion

In summary, we have demonstrated a self‐multiplexing SSOECT based on the in‐electrolyte computing concept. The devices underwent impedance analysis, revealing an Ohmic‐like resistance change in the solid electrolyte with varying CE‐WE distances. These variations in R_b_ correspond to distance‐modulated ionic doping of the conjugated semiconductor. Consequently, I_d_ exhibited a distance‐dependent effect in both transfer and transient behavior of the SSOECT due to shifts in V_eff_ and τ, facilitating input signal identification. This behavior was further elucidated through in situ UV–vis–NIR spectrochemical studies, which showed a more pronounced and faster shift in the absorption spectra for CE1. We also explored the parameters affecting the speed and modulation ratio of the multiplexer, including ionic liquid concentration, channel dimension, measurement protocol, and channel materials. Our findings indicate that reducing the ionic liquid content or increasing the channel dimension can enhance the modulation ratio (Δ*R*/R_0_). Additionally, shortening the pulse width (PW) can maximize Δ*R*/R_0_, provided that signals from the remote gate can be detected. Solid electrolytes demonstrated a substantial ion modulation effect, with a ≈402‐fold modulation ratio in I_d_ and 100% addressing accuracy for 12 input signals. The multiplexer was further integrated with a 3 × 4 tactile sensor array, enabling recognition of multi‐input bio‐signals using a single polymeric channel without additional circuitry. This approach significantly reduces the use of electrical components and associated costs. We believe this OECT‐based multiplexing platform will facilitate the implementation of more complex learning rules in the future without increasing circuit complexity, such as second‐order neuromorphic computing.^[^ ^41^ ^]^

## 4 Experimental Section

### Materials

Poly(3,4‐ethylenedioxythiophene):poly(styrenesulfonate) (PEDOT:PSS, Clevios PH1000) was purchased from Heraeus. Poly(3‐hexythiophene‐2,5‐diyl) (P3HT), poly[(5‐fluoro‐2,1,3‐benzothiadiazole‐4,7‐diyl) (4,4‐dihexadecyl‐4H‐ cyclopenta [2,1‐b:3,4‐b'] dithiophene‐2,6‐diyl) (6‐fluoro‐2,1,3‐benzothiadiazole‐4,7‐diyl) (4,4 dihexadecyl‐4H cyclopenta [2,1‐b:3,4‐ b']dithiophene‐2,6‐diyl)] (PCDTFBT), thermoplastic polyurethane (TPU), 1‐ethyl‐3‐methylimidazolium bis(trifluoromethylsulfonyl)amide (EMIM:TFSI), and all the processing solvents including acetone, dimethylformamide, and chloroform were all obtained from Sigma–Aldrich and used as received.

### Fabrication of OECT‐Based Multiplexer

First, Si/SiO_2_ wafer was cleaned with acetone and ethanol for 5 min each in ultrasonication. AZ5214E photoresist was used for source/drain/gate patterning, which was spin‐coated on top of the pre‐cleaned substrates and exposed to UV light using a SUSS MJB4 mask aligner and then developed by AZ developer. Thereafter, 50 nm gold with 5 nm titanium as a seed layer was evaporated on the substrate sequentially via E‐beam evaporation, followed by lift‐off of the photoresist by immersion of the substrates in acetone for 10 min with sonication. Then, a 1.4 µm thick Parylene C layer with an adhesion promoter of 3 (trimethoxysilyl)propyl methacrylate (A‐174 Silane) was deposited to insulate the contacts from the electrolyte using SCS Labcoater. An anti‐adhesion layer of dilute cleaner (Micro‐90, 10 wt.% in deionized water) was spin‐coated to facilitate the peel‐off procedure. After that, a second Parylene‐C layer (≈2 µm) was deposited, acting as a sacrificial layer. The substrates were subjected to a second lithography process to expose the channel, gate, and contact pad area, where AZ4620 and AZ developer were used as photoresist and developer, respectively. The patterned channel, gate, and contact pad areas were etched via reactive ion etching (Oxford Plasmalab80) at 50 sccm O2, 10 sccm CHF3, and 160 W operating conditions. wt.% (1.3) PEDOT:PSS dissolved in water, 5 mg mL^−1^ P3HT and PCDTFBT dissolved in chloroform were spin‐coated on the prepared substrate, followed by peeling‐off of the 2nd layer parylene. For the preparation of solid electrolyte, 2 g TPU pellet was dissolved in 4 mL acetone and 4 mL dimethylformamide cosolvent, followed by vigorous stirring at 100 °C for 6 h until all pellets dissolved. Then, the ionic liquids were added to the above solution with varying weight ratios, followed by vigorous vibration for 30 min. The solid electrolyte solution was directly spin‐coated on top of the channel/gates and dried in a vacuum for more than 24 h.

### Electrical Characterizations

The *I*–*V* characteristics of the films and all the OECT device characteristics, including output and transfer curves and pulse measurement, were measured under ambient conditions using Keysight precision source/measure unit (B2912A) and a probe station (Karl Suss PM5). The scan rate was kept at 50 mV s^−1^ for DC.

### Electrochemical Impedance Spectroscopy (EIS)

EIS was employed to investigate the shift in R_b_ of solid electrolyte films using the Metrohm Autolab system. The input signal was a sine wave (amplitude of 50 mV) with a frequency range from 1 000 000 to 100 Hz. Si/SiO_2_ substrate was patterned with AZ5214 and AZ developer. Thereafter, 50 nm gold was evaporated on the substrate sequentially via E‐beam evaporation, which was followed by lift‐off. Then, PCDTFBT solution was spin‐coated on the prepared substrate, and the area of the semiconductor was patterned with a cotton swab, which was followed by spin‐coating of the solid electrolyte.

### Electrochemistry Spectroscopy

ITO/glass was patterned with AZ4620 and AZ developer. Then, the substrate was soaked in HCl (37%) for 12 min to remove the exposed ITO, which was followed by lift‐off of the photoresist. Thereafter, PCDTFBT solution was spin‐coated on the substrate, and the area of the semiconductor was patterned with a cotton swab. The solid electrolyte solution was then spin‐coated on the substrate, which was confined by a pillar made of PI tape. A Keysight precision source/measure unit (B2912A) was chosen to apply biasing between the gate/source. The absorption spectra were recorded using a UV–vis–NIR spectrophotometer (Ocean Optics). Another sample without the semiconductor layer was used to obtain the baseline.

### Fabrication of Analog Pressure Sensor

SEBS was poured and dried onto a micro‐patterned mold, which was followed by peeling off and spin‐coating with PEDOT:PSS as the sensing layer. The base of the sensor was printed with silver ink by a PCB printer on PI substrate. And the device was encapsulated with two layers of PET films.

### Fabrication of Tactile Sensor Array

The base of the sensor was printed with silver ink by a PCB printer on PI substrate, while the sensing layer was fabricated by evaporating a 100 nm‐thick gold film on a PI substrate through a shadow mask.

## Conflict of Interest

The authors declare no conflict of interest.

## Supporting information



Supporting Information

Supplemental Video 1

Supplemental Video 2

Supplemental Video 3

Supplemental Video 4

## Data Availability

The data that support the findings of this study are available from the corresponding author upon reasonable request.
