# Supplementary material for: Exploiting Spatial Ionic Dynamics in Solid‐State Organic Electrochemical Transistors for Multi‐Tactile Sensing and Processing
Source: Adv Sci (Weinh). 2024 Sep 27;11(43):2405902. doi: 10.1002/advs.202405902 (PMC11578318; doi:10.1002/advs.202405902)
Supplement: Supplementary file 1 — Supporting Information [file ADVS-11-2405902-s004.docx]

**Exploiting spatial ionic dynamics in solid-state organic electrochemical transistors for multi-tactile sensing and processing**

Kunqi Hou^1^, Shuai Chen^2^, Rohit Abraham John^3^, Qiang He^2^, Zhongliang Zhou^2^, Nripan Mathews^4^, Wen Siang Lew^1*^ and Wei Lin Leong^2*^

*^1^School of Physical and Mathematical Sciences, Nanyang Technological University*

*21 Nanyang Link, 637371 Singapore. E-mail:* [*wensiang@ntu.edu.sg*](mailto:wensiang@ntu.edu.sg)

*^2^School of Electrical and Electronic Engineering, Nanyang Technological University 50 Nanyang Avenue, 639798 Singapore. E-mail:* [*wlleong@ntu.edu.sg*](mailto:wlleong@ntu.edu.sg)

*^3^Laboratory of Inorganic Chemistry, Department of Chemistry and Applied Biosciences, ETH Zürich, CH-8093 Zürich, Switzerland.*

*^4^School of Materials Science and Engineering, Nanyang Technological University*

*50 Nanyang Avenue, 639798 Singapore.*


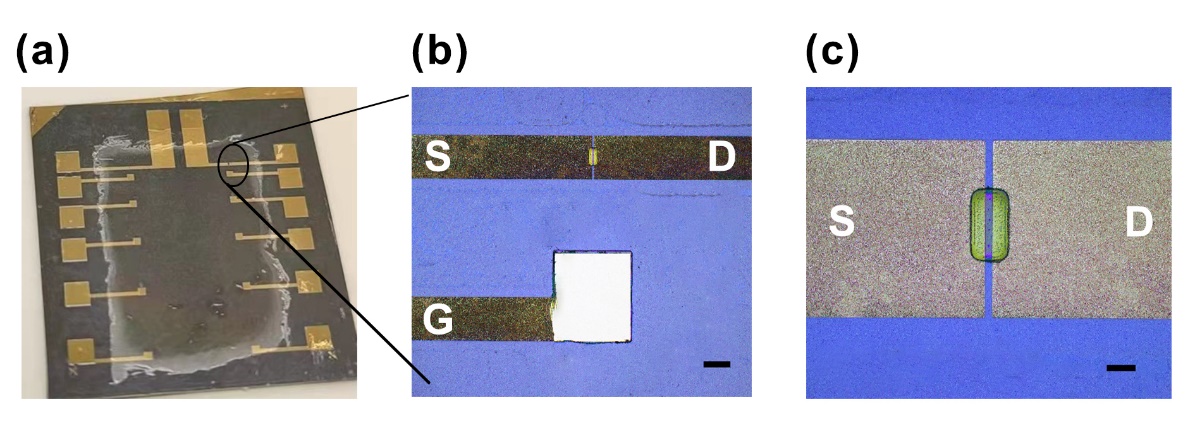


Figure S1. a, b) Optical images of the fabricated multiplexer device, where scale bar in b is 200 μm. c) Optical image of the spin-coated semiconducting channel, where scale bar represents 40 μm.


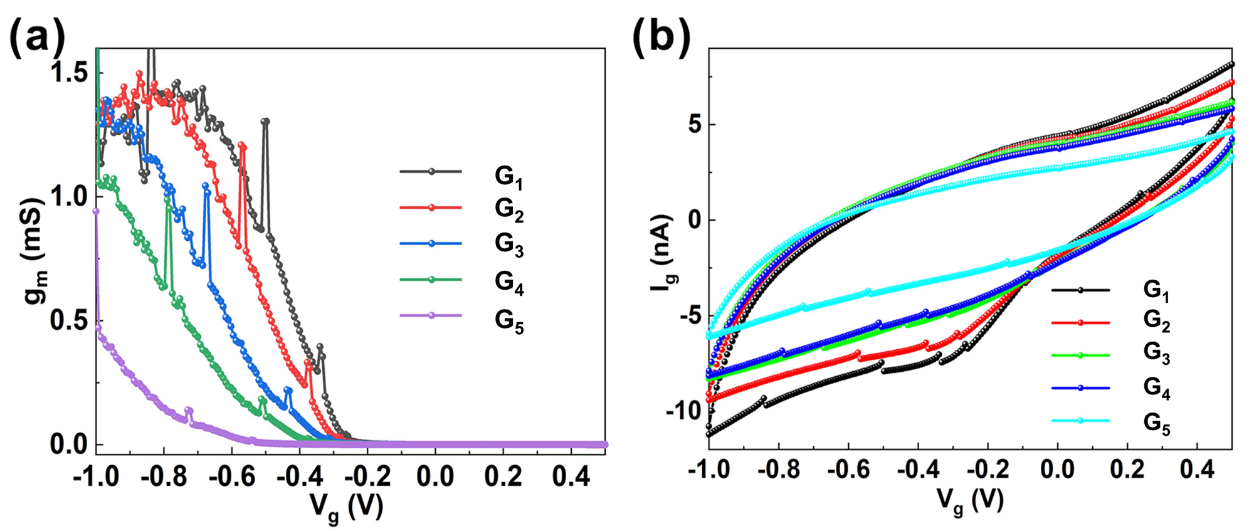


Figure S2. a) Transconductance value of transistor for each gate with varying spatial dynamics. b) Gate-source current of transistor for each gate with varying spatial dynamics.


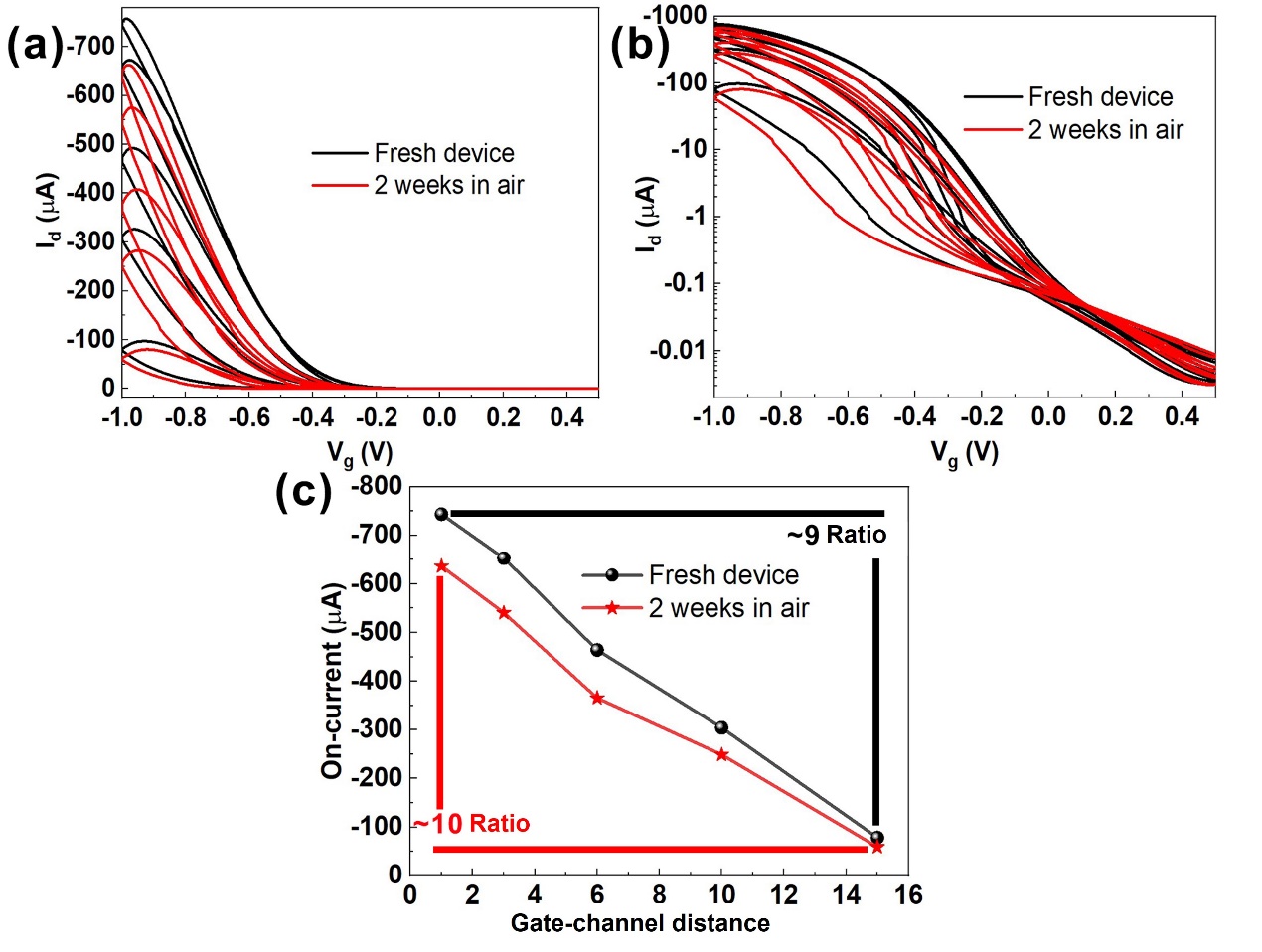


Figure S3. Long-term stability of SSOECT-based multiplexer. Transfer curves of fresh and device after 2 weeks in linear (a) and semi log (b) plot. c) On-current modulating ratio of fresh and device after 2 weeks.


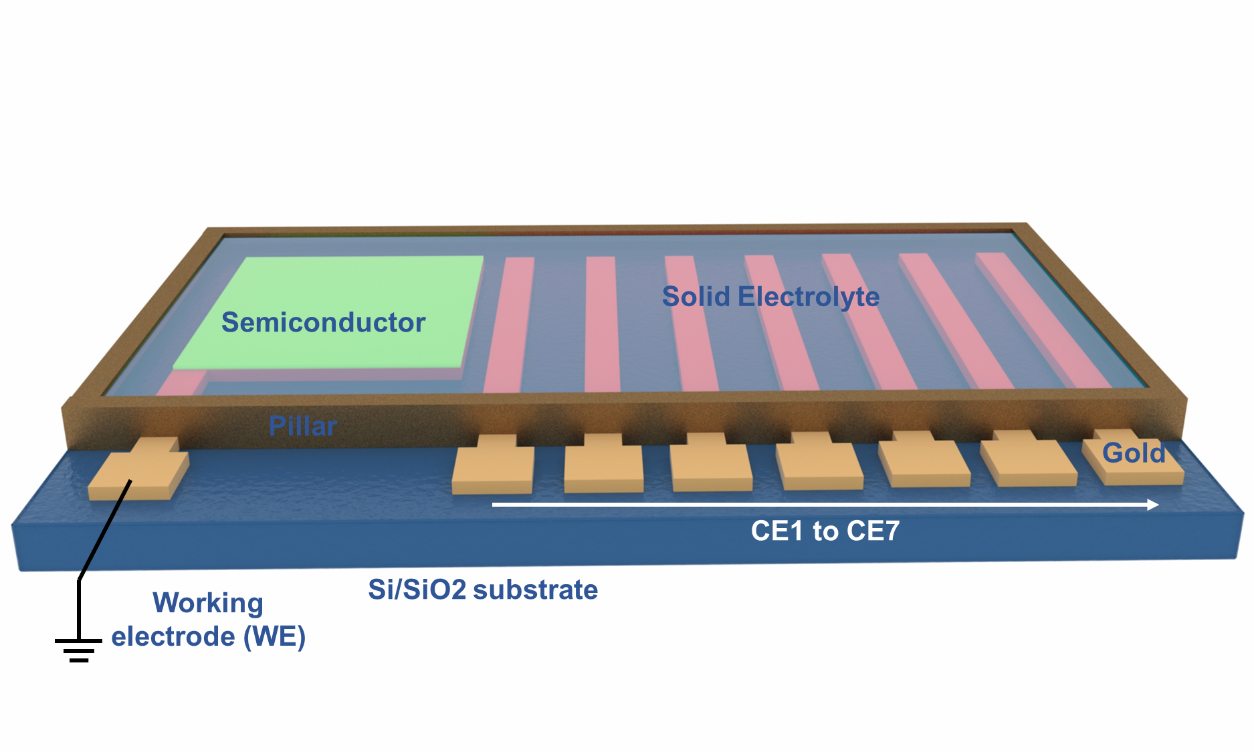


Figure S4. Schematic illustration of EIS substrate. The areas of WE are 0.6 mm * 0.3 mm, for CEs is 0.8 mm * 0.1 mm. The distances between different CEs to WE are 2 mm, 5 mm, 8 mm, 11 mm, 14 mm, 17 mm, and 20 mm, respectively.


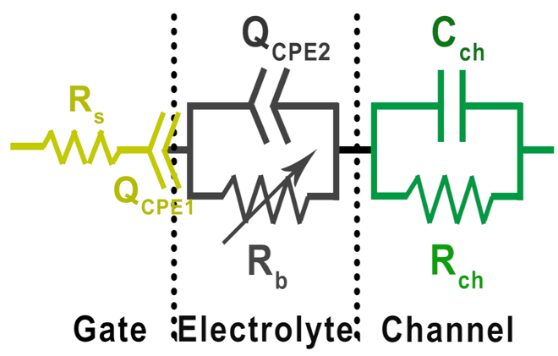


Figure S5. Equivalent circuit from gate to source terminal, where electrolyte bulk resistance is alternated by gate-channel distance. R_s_ stands for the series resistance generated by wire connections/instrument internal resistance. When a negative bias is applied on a gate (G_n_), cations inside the electrolyte move toward the electrode interface, generating a compact Helmholtz layer, which can be demonstrated by a capacitor in series (Q_CPE1_).^1^ Anions move into the semiconductor layer at the same time, which can be described as a capacitor (C_ch_) in parallel with a resistor (R_ch_), where R_ch_ is typically high and can be neglected.^2,3^ Ion movement inside the solid electrolyte can be modelled as a bulk ionic resistor (R_b_) in parallel with an ionic capacitor (Q_CPE2_), where R_b_ of the electrolyte can be tuned and presents a positive proportion with the distance between the counter and reference electrodes.^4,5^


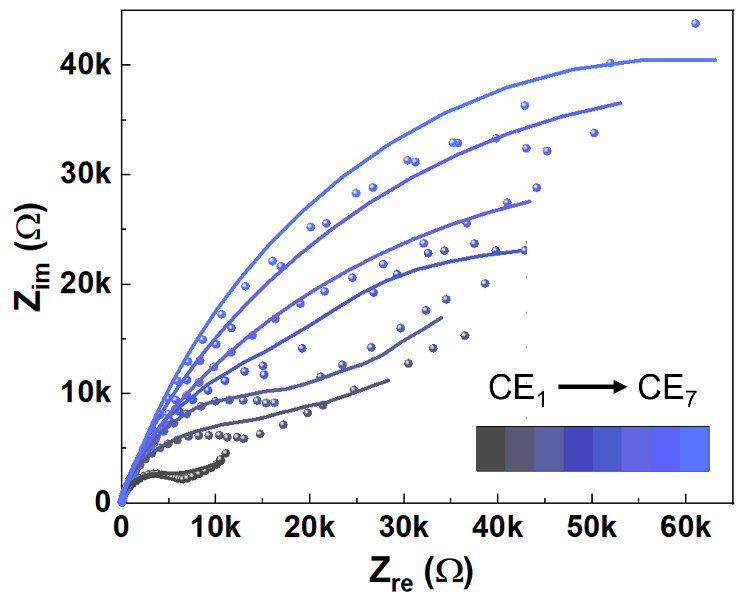


Figure S6. Nyquist plot for varying CE-WE distances. Measured data are shown by scatters, while the fitted curves using the equivalent circuit are shown by solid lines.


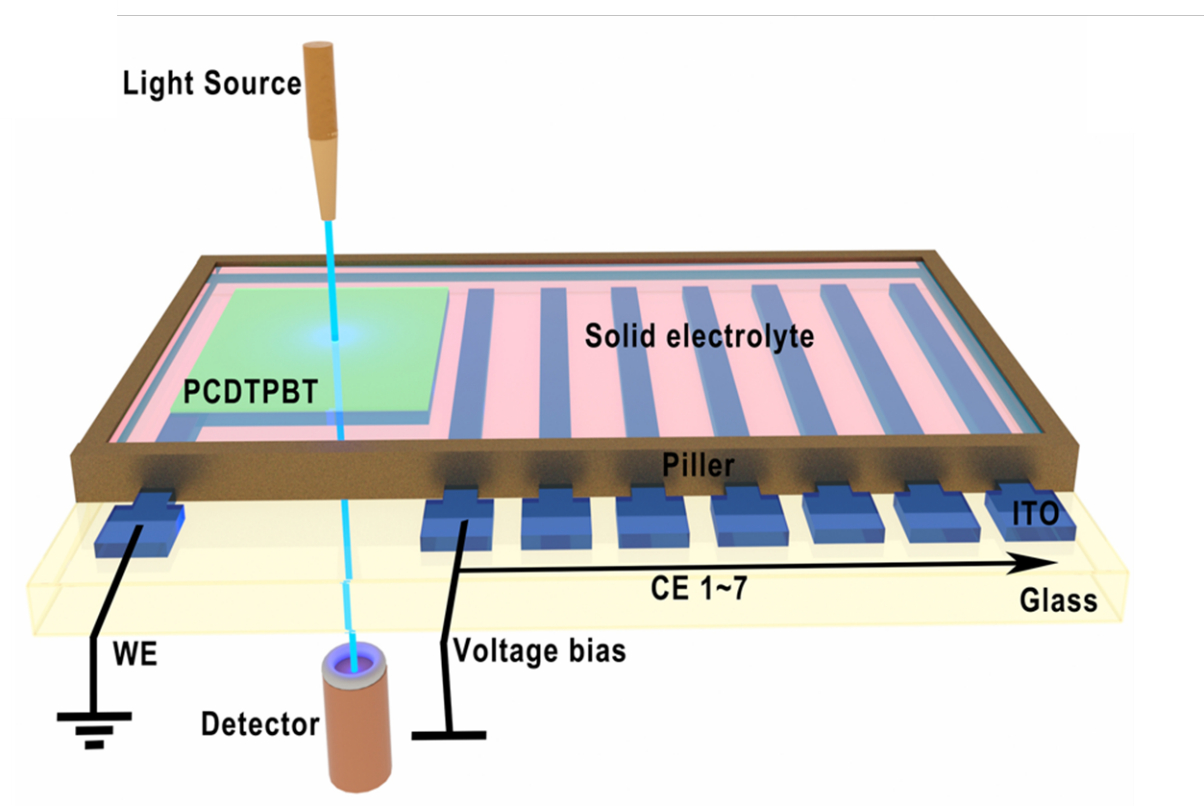


Figure S7. Schematic illustration of the sample during UV-vis-NIR measurement. The distances between different CEs to WE are 2 mm, 5 mm, 8 mm, 11 mm, 14 mm, 17 mm, and 20 mm, respectively. The solid electrolyte covers the whole semiconductor film and CE areas, which is confined by a pillar.


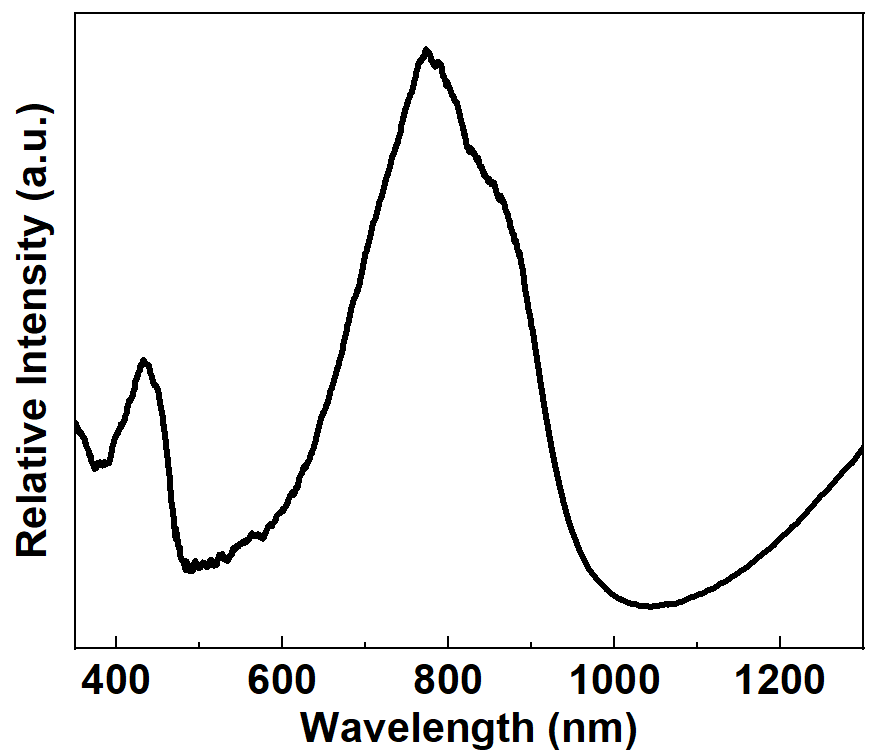


Figure S8. Absorption spectra of pristine PCDTFBT film on ITO/glass substrate.


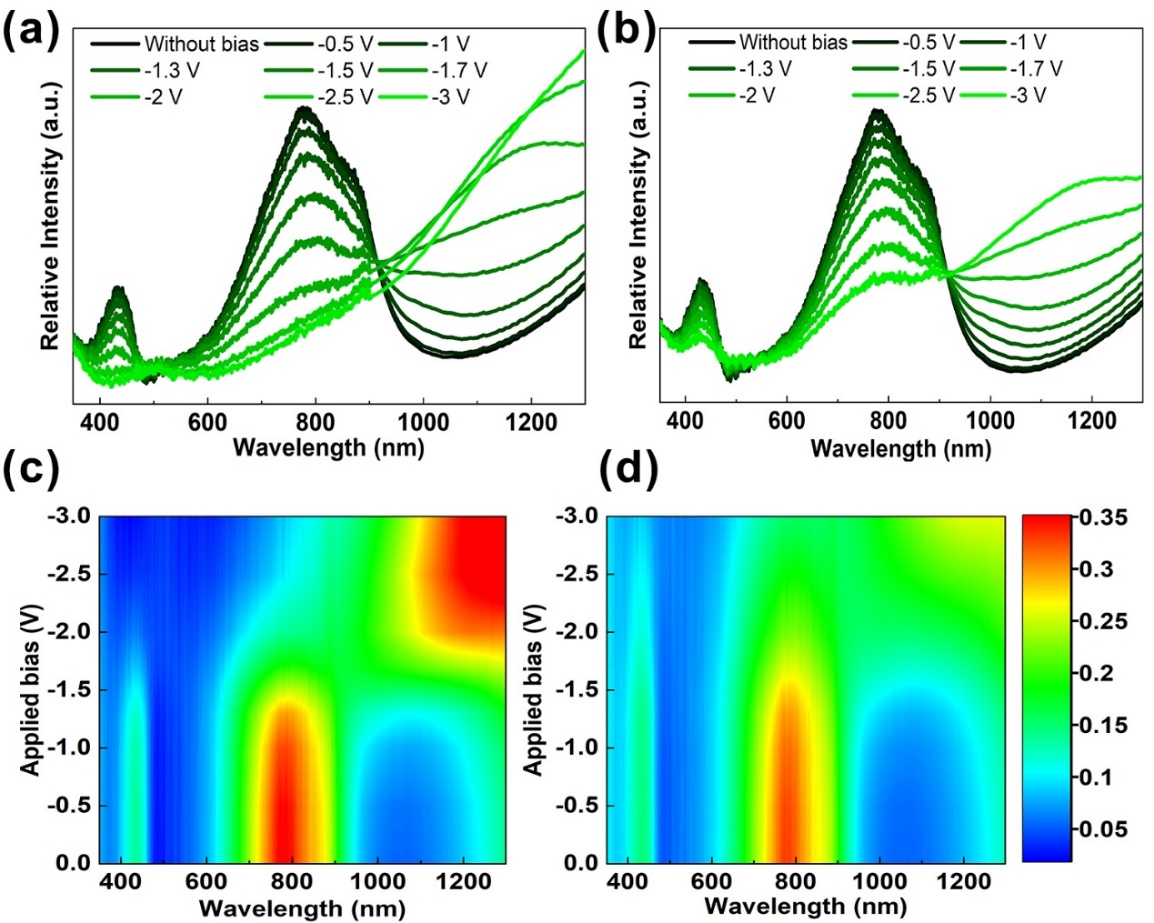


Figure S9. a, b) Detailed absorption spectra of PCDTFBT film upon applying voltage bias on WC2mm (a) and WC20mm (b). c, d) 3D color map of absorption intensity of PCDTFBT film under voltage bias on WC2mm (c) and WC20mm (d), where a larger tuning range for WC2mm is observed.


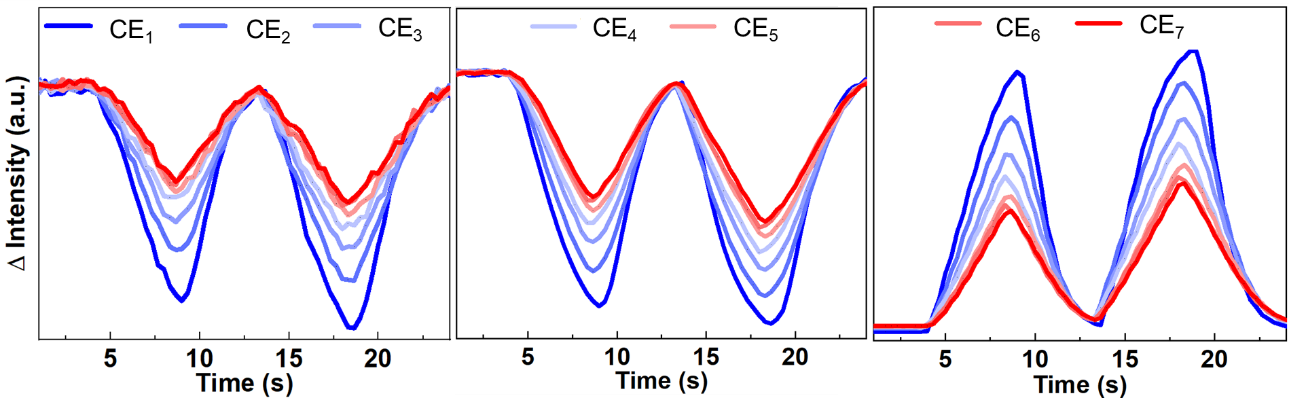


Figure S10. Dynamic changes in relative intensity at the characteristic wavelengths of 432 nm, 772 nm, and 1200 nm under a voltage pulse with 5 s/5 s delay time/pulse width and 2 V/-3 V base/pulse value.


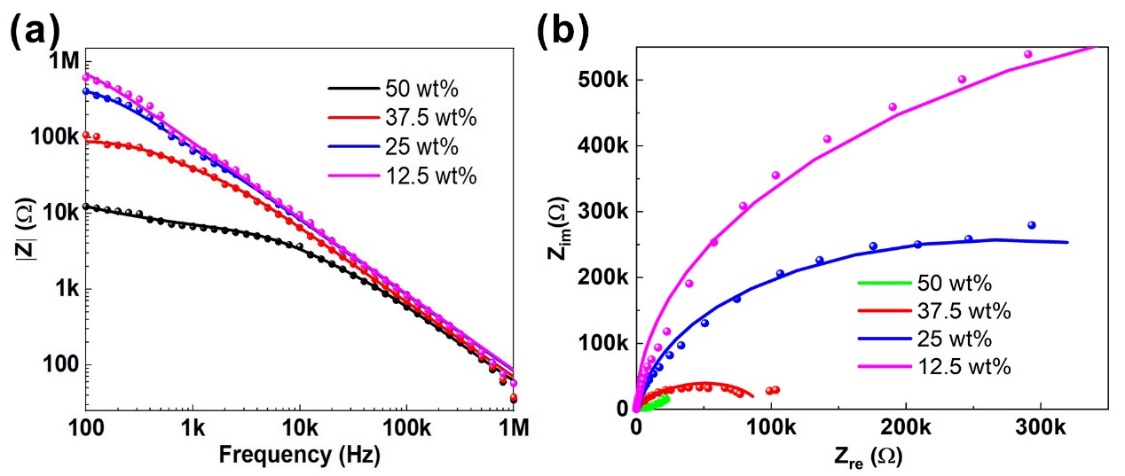


Figure S11. a) Amplitude of impedance spectrum (|Z| vs frequency) and b) Nyquist plot (Z_re_ vs Z_im_) of solid electrolytes in varying concentrations. Measured data are shown by scatters, while the fitted curves using equivalent circuit are shown by solid lines.


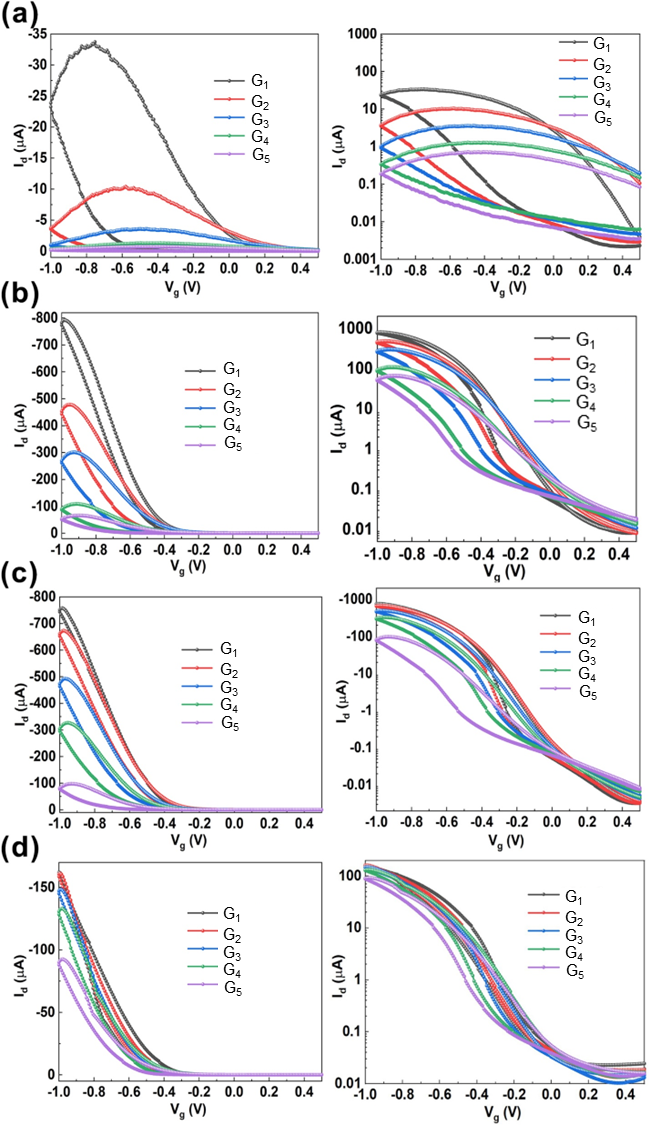


Figure S12. Transfer performance in linear and log scale of multiplexers with varying solid electrolyte concentrations. 12.5, 25, 37.5, and 50 wt% for a), b), c), and d), respectively.


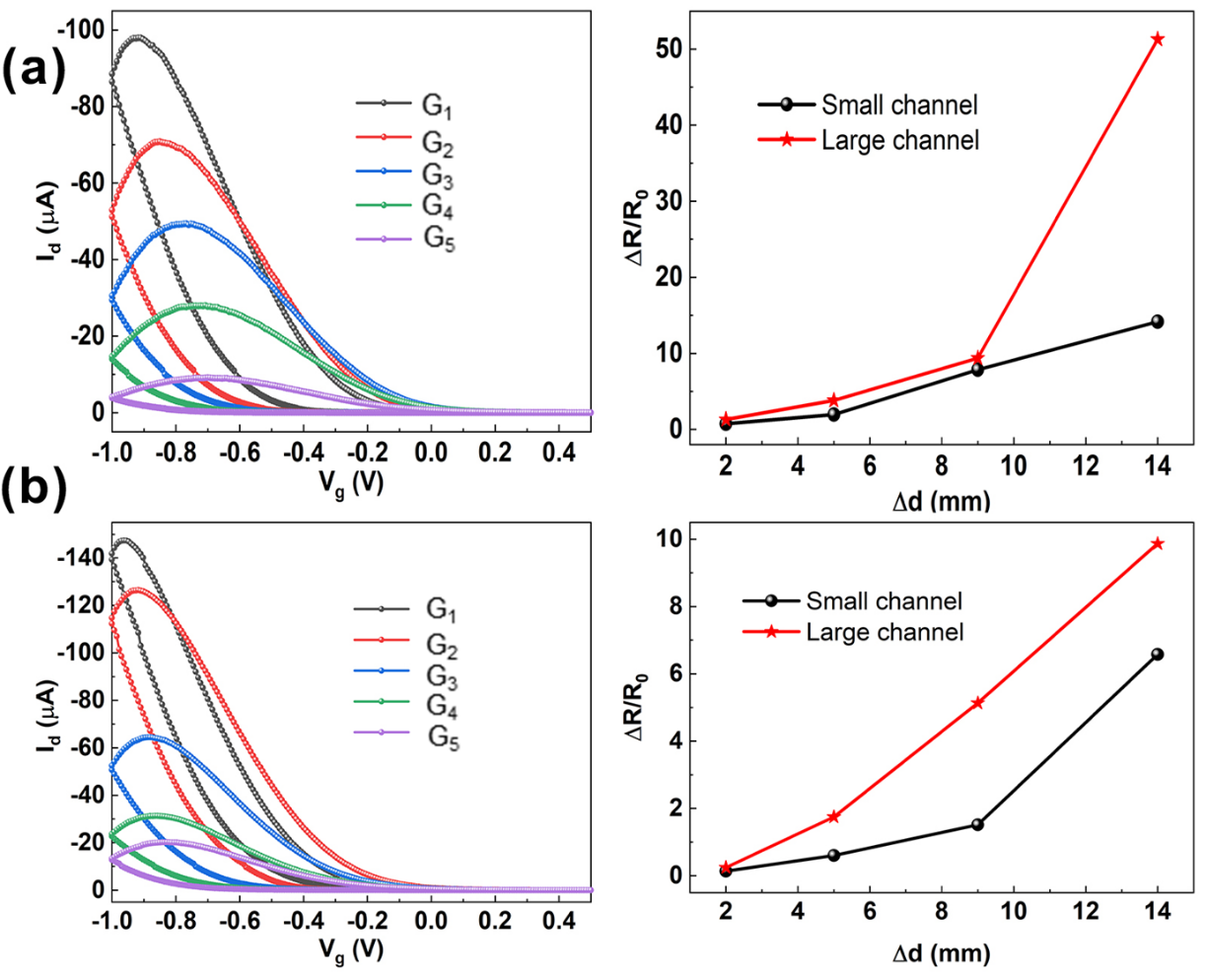


Figure S13. Transfer performance for large channel 200 um/50 um using 25 wt% (a), and 37.5 wt% (b) solid electrolyte and their corresponding ΔR/R_0_.


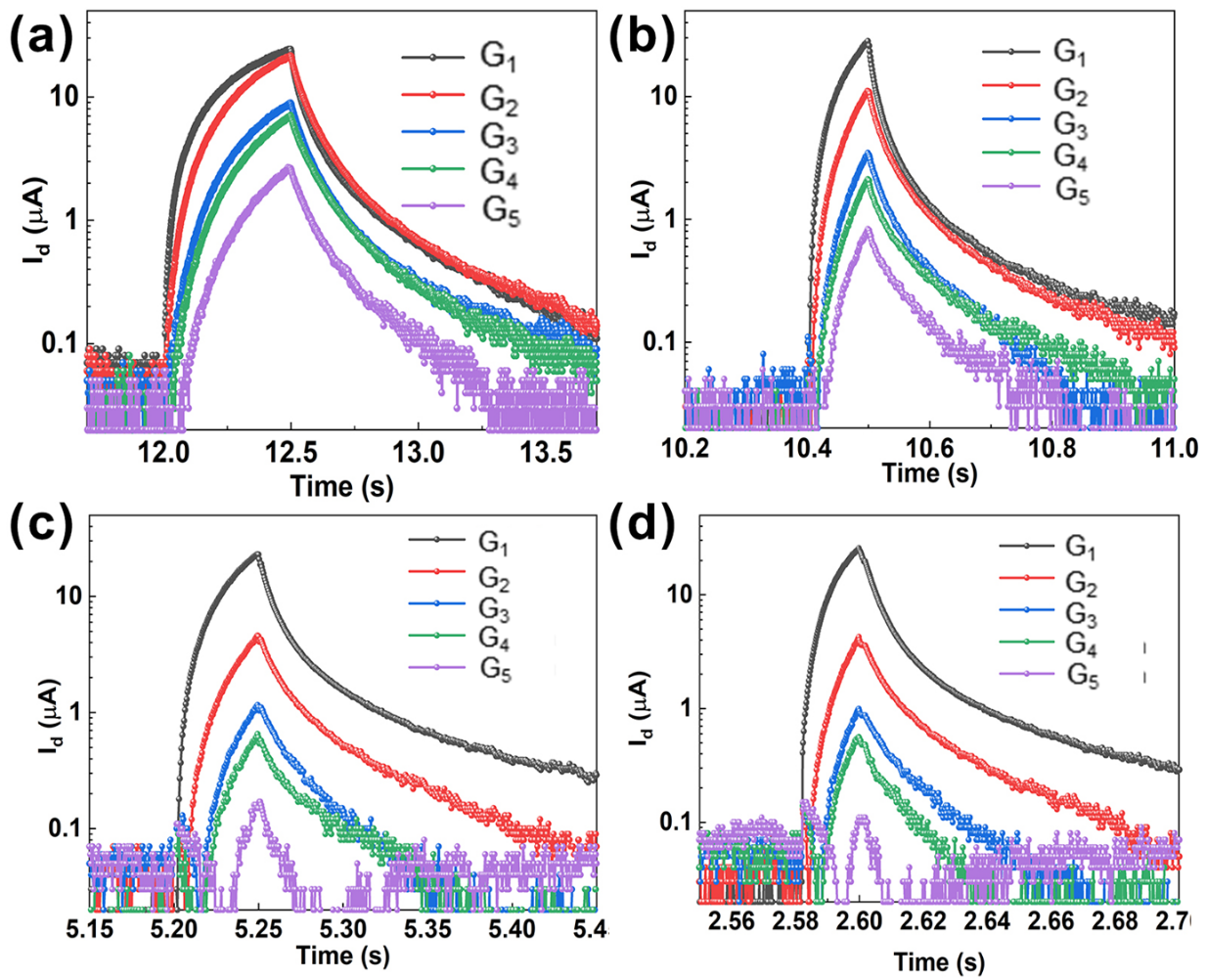


Figure S14. Transient response of gates with varying spatial dynamics under 500 ms (a), 100 ms (b), 50 ms (c), 20 ms (d) PW.


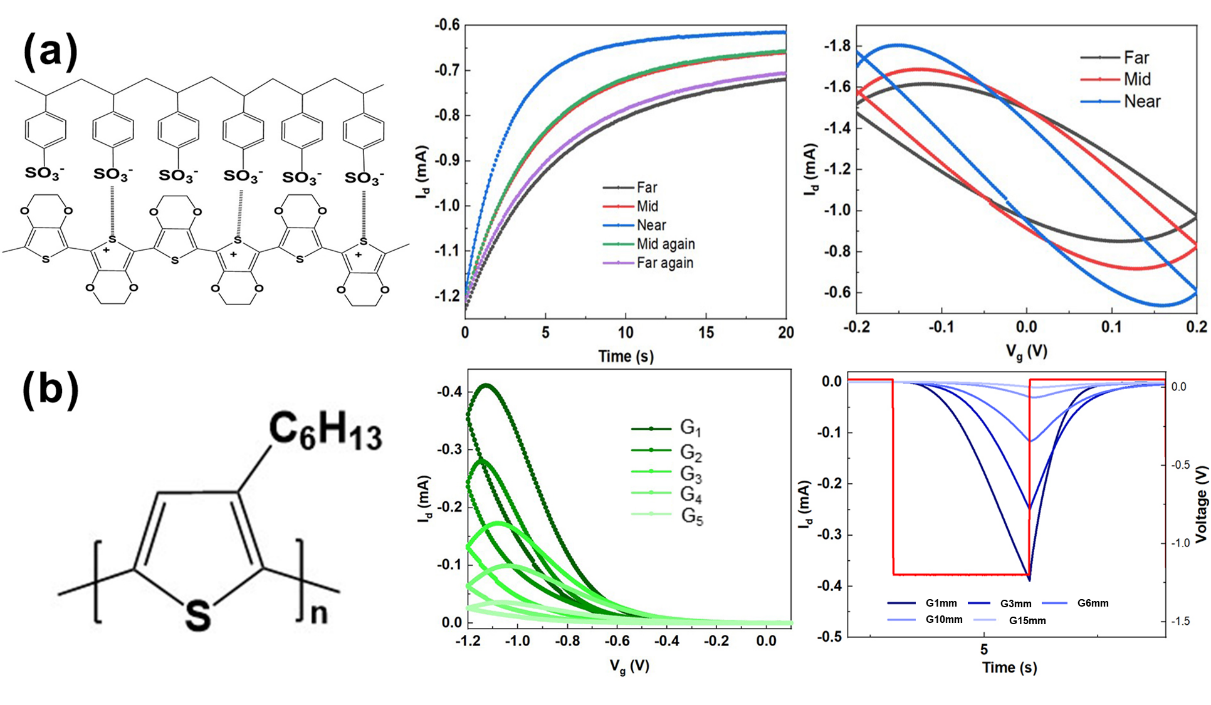


Figure S15. Exploring the accessibility of such multiplexing technique. a) Chemical structure (left), output behavior (middle), and transfer curves (right) of PEDOT:PSS-base self-multiplexing SSOECT. b) Chemical structure (left), transfer curves (middle), and transient behavior (right) of P3HT-base self-multiplexing SSOECT.


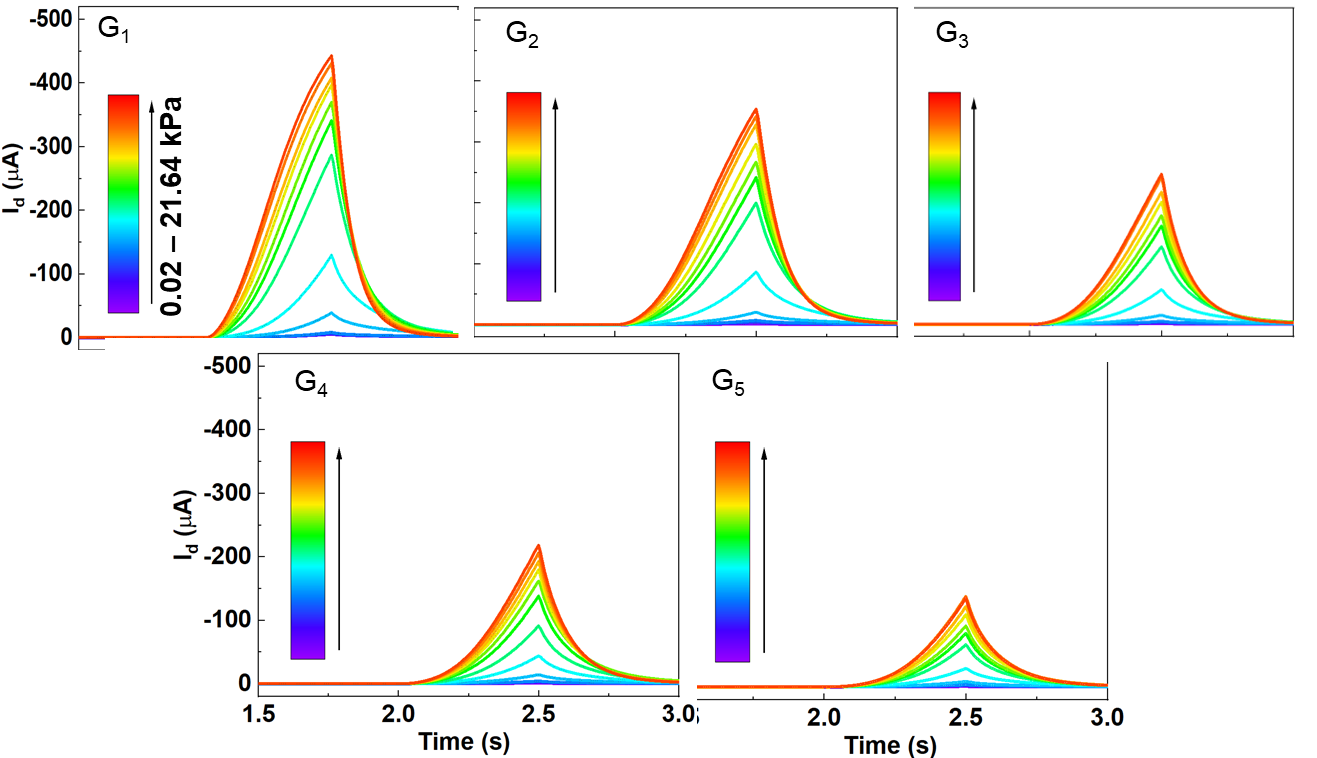


Figure S16. Transfer/transient behavior of the integrated systems from 0.02 – 21. 64 kPa, -1.5 V/-0.5 V incident pulse/I_d_ applied.


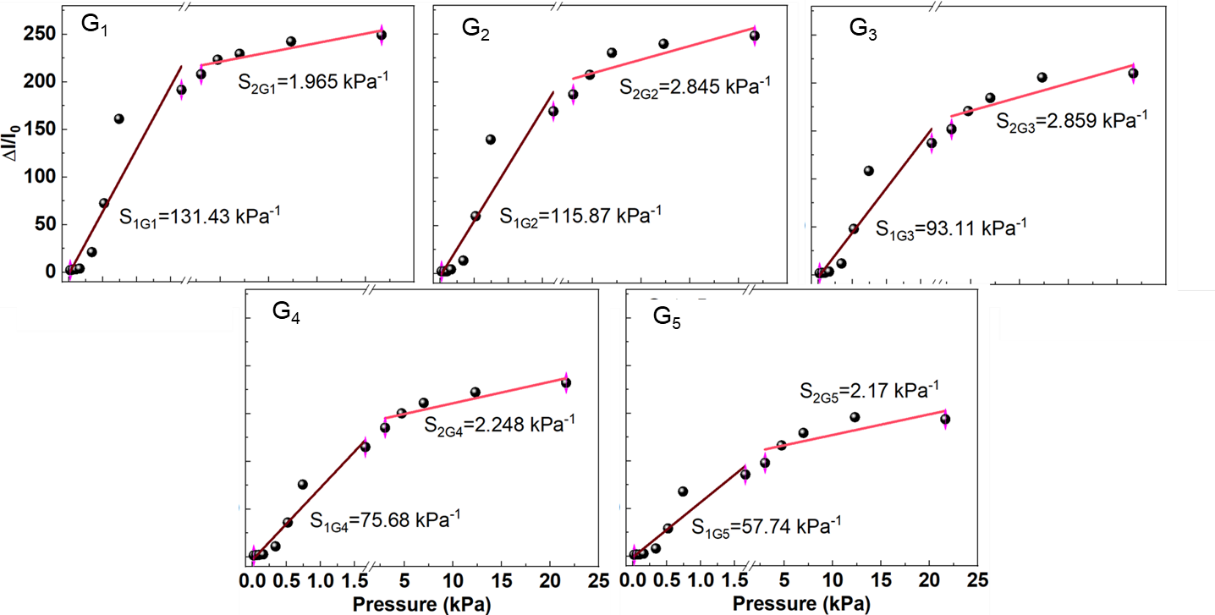


Figure S17. Comparison of the sensitivities in the low and high-pressure region when the pressure sensor connected with G1mm to G15mm (Single consecutive measurement).


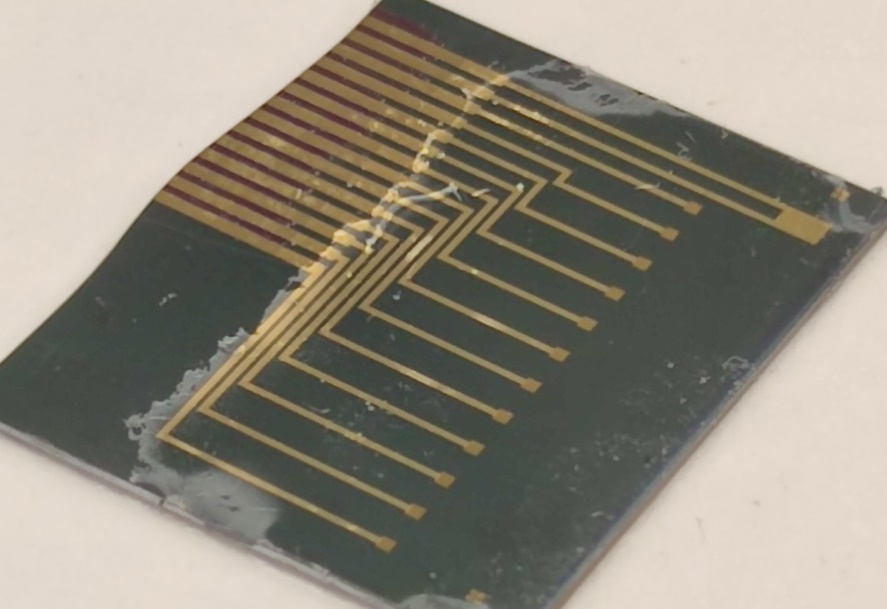


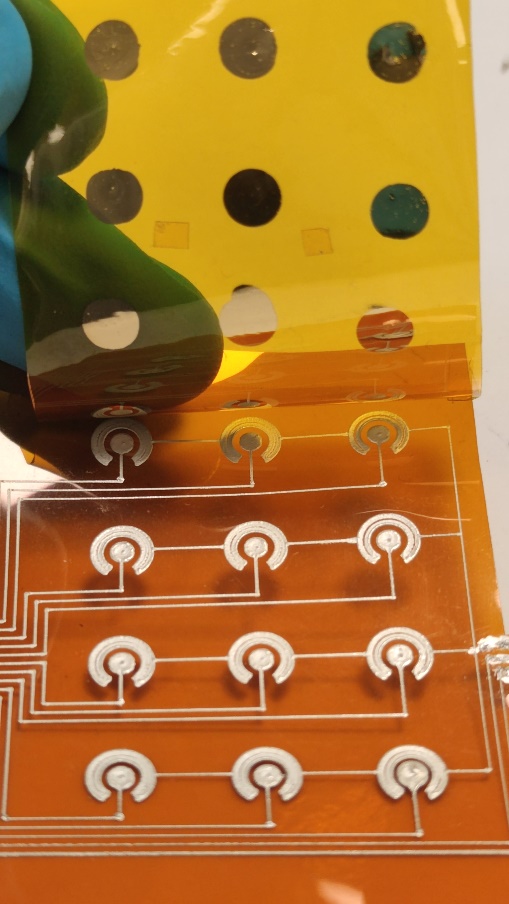


Figure S18. Optical images of 12-gate self-multiplexing SSOECT (up), and haptic sensor array (bottom).


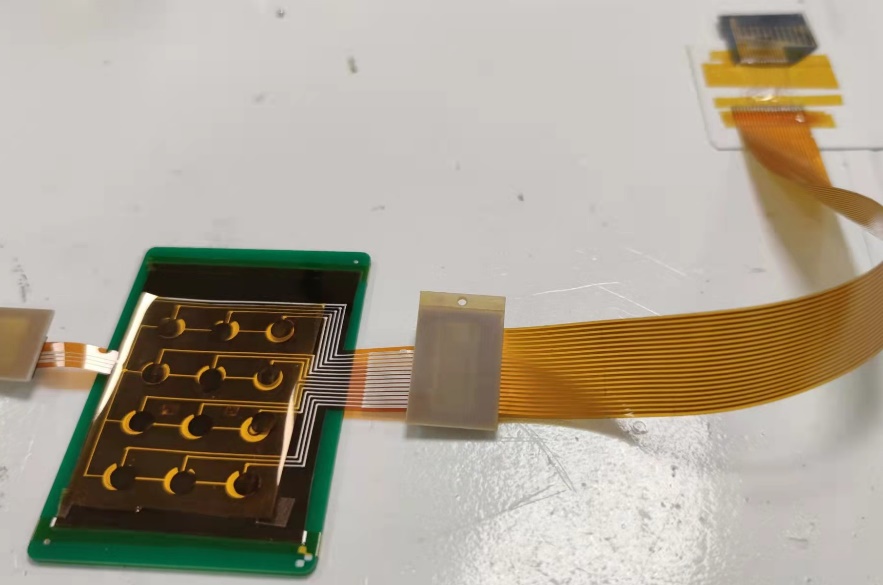


Figure S19. Optical image of SSOECT-based multiplexer-haptic sensor array integrated system.


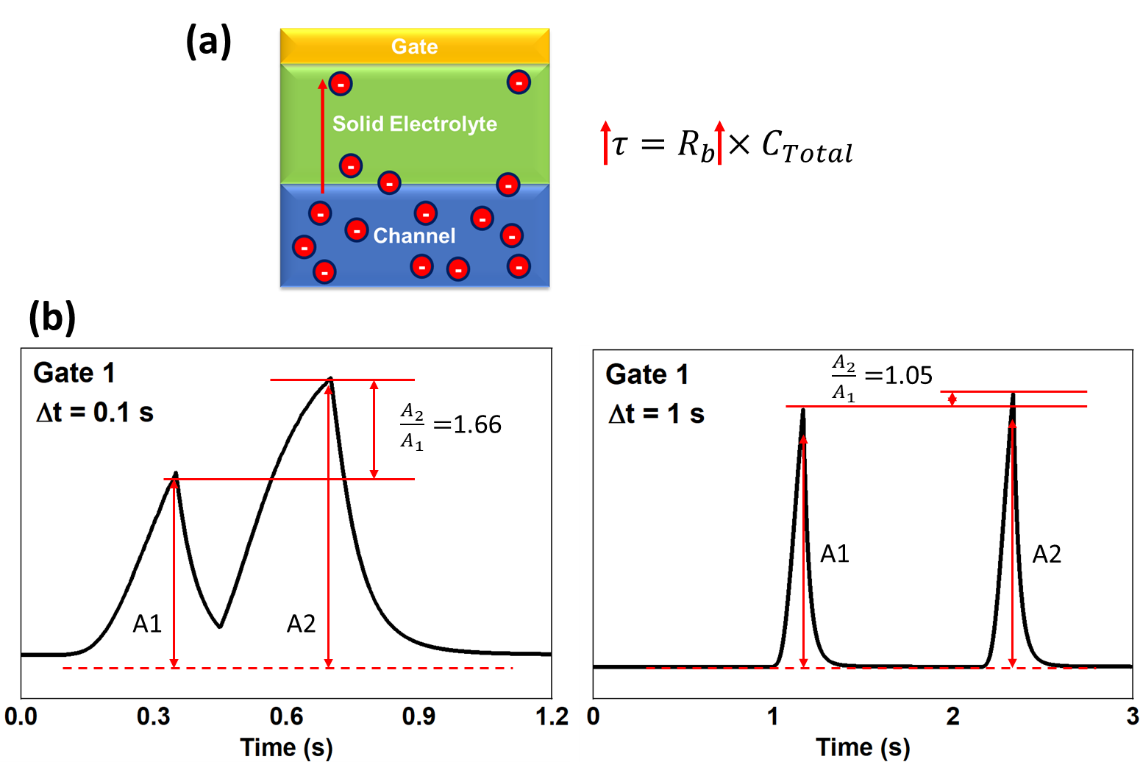


Figure S20. a) Schematic demonstration on recovery time of dopant ions. b) PPF characterization of SSOECT with 37.5% solid electrolyte.


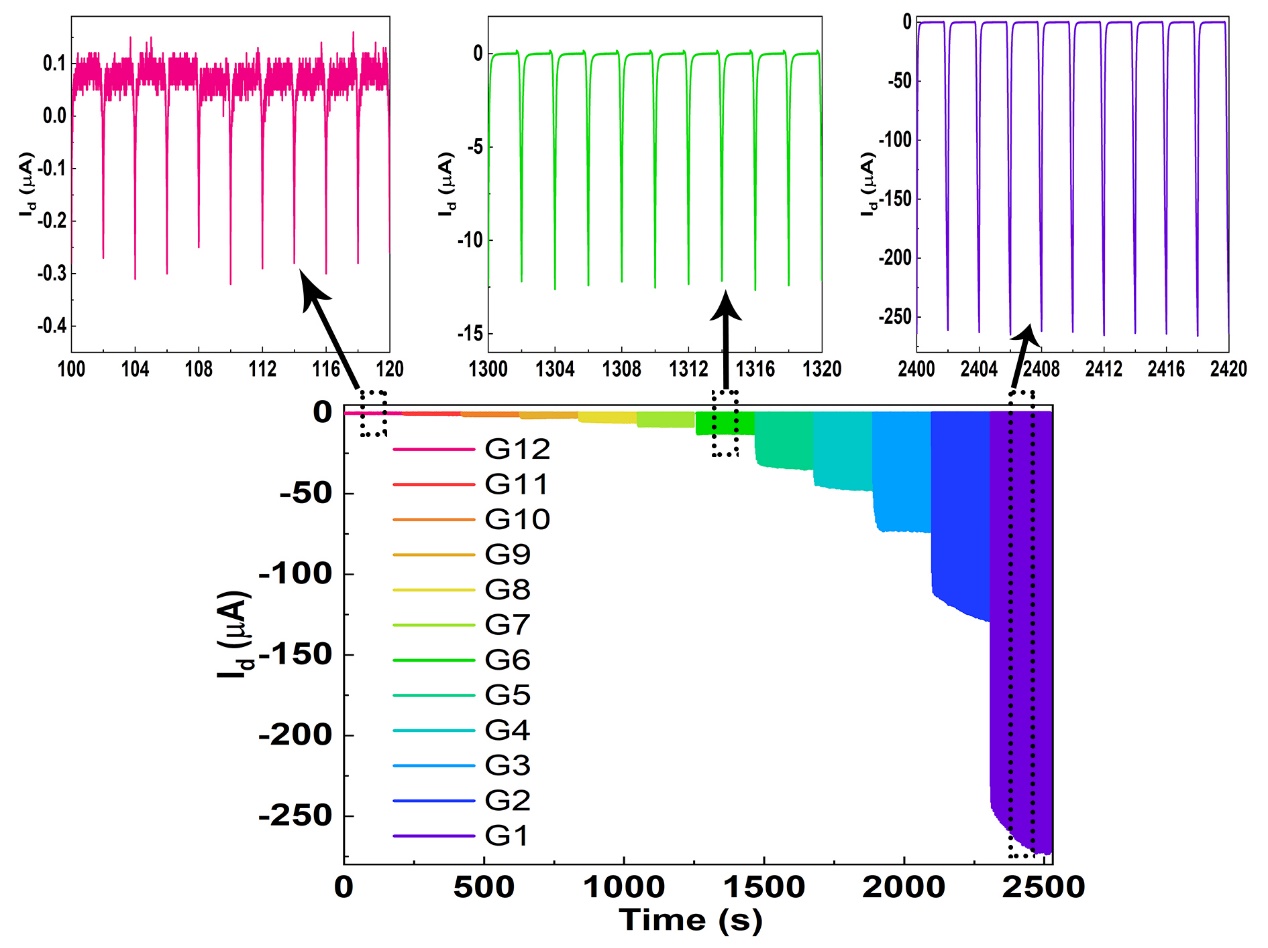


Figure S21. Pulsing stability of multiplexer. One hundred pulses were applied from G_12_ to G_1_ sequentially with 0.3/-1.6 V base/pulse amplitude, and 1.7/0.3 s delay/pulse width.

**Reference.**

1 Bard, A. J., Faulkner, L. R. & White, H. S. *Electrochemical methods: fundamentals and applications*. (John Wiley & Sons, 2022).

2 Mariani, F., Gualandi, I., Tessarolo, M., Fraboni, B. & Scavetta, E. PEDOT: dye-based, flexible organic electrochemical transistor for highly sensitive pH monitoring. *ACS applied materials & interfaces* **10**, 22474-22484 (2018).

3 Rivnay, J. *et al.* High-performance transistors for bioelectronics through tuning of channel thickness. *Science Advances* **1**, e1400251, doi:10.1126/sciadv.1400251 (2015).

4 Bérardan, D., Franger, S., Meena, A. & Dragoe, N. Room temperature lithium superionic conductivity in high entropy oxides. *Journal of Materials Chemistry A* **4**, 9536-9541 (2016).

5 Arof, A., Amirudin, S., Yusof, S. & Noor, I. A method based on impedance spectroscopy to determine transport properties of polymer electrolytes. *Physical Chemistry Chemical Physics* **16**, 1856-1867 (2014).
